# AP-1 Inhibition by SR 11302 Protects Human Hepatoma HepG2 Cells from Bile Acid-Induced Cytotoxicity by Restoring the NOS-3 Expression

**DOI:** 10.1371/journal.pone.0160525

**Published:** 2016-08-04

**Authors:** Sandra González-Rubio, Clara I. Linares, Patricia Aguilar-Melero, Manuel Rodríguez-Perálvarez, José L. Montero-Álvarez, Manuel de la Mata, Gustavo Ferrín

**Affiliations:** 1 Maimonides Institute of Biomedical Research in Córdoba (IMIBIC), Reina Sofía University Hospital, University of Córdoba, Córdoba, Spain; 2 Biomedical Research Centre Network, Digestive and Liver Diseases (CIBEREHD), Instituto de Salud Carlos III, Córdoba, Spain; University of Navarra School of Medicine and Center for Applied Medical Research (CIMA), SPAIN

## Abstract

The harmful effects of bile acid accumulation occurring during cholestatic liver diseases have been associated with oxidative stress increase and endothelial nitric oxide synthase (NOS-3) expression decrease in liver cells. We have previously reported that glycochenodeoxycholic acid (GCDCA) down-regulates gene expression by increasing SP1 binding to the NOS-3 promoter in an oxidative stress dependent manner. In the present study, we aimed to investigate the role of transcription factor (TF) AP-1 on the NOS-3 deregulation during GCDCA-induced cholestasis. The cytotoxic response to GCDCA was characterized by 1) the increased expression and activation of TFs cJun and c-Fos; 2) a higher binding capability of these at position -666 of the NOS-3 promoter; 3) a decrease of the transcriptional activity of the promoter and the expression and activity of NOS-3; and 4) the expression increase of cyclin D1. Specific inhibition of AP-1 by the retinoid SR 11302 counteracted the cytotoxic effects induced by GCDCA while promoting NOS-3 expression recovery and cyclin D1 reduction. NOS activity inhibition by L-NAME inhibited the protective effect of SR 11302. Inducible NOS isoform was no detected in this experimental model of cholestasis. Our data provide direct evidence for the involvement of AP-1 in the NOS-3 expression regulation during cholestasis and define a critical role for NOS-3 in regulating the expression of cyclin D1 during the cell damage induced by bile acids. AP-1 appears as a potential therapeutic target in cholestatic liver diseases given its role as a transcriptional repressor of NOS-3.

## Introduction

Bile acids (BAs) are physiological detergents synthesized from cholesterol in the liver, which are required for absorption and transport of dietary fats and lipid-soluble vitamins, and disposal of toxic metabolites, drugs and xenobiotics. Cholestatic liver diseases (CLD) include a group of disorders with different epidemiology, pathophysiology, clinical course and prognosis, sharing a defective BA formation and/or transportation from the liver to the intestine. Cholestasis may produce accumulation of toxic BAs in liver leading to hepatocellular apoptosis, which represents a key cellular mechanism for BA-mediated liver injury and progression of CLD towards fibrosis, cirrhosis and chronic liver failure [1] [2] [3]. Thus, the consequences of untreated cholestasis are severe and increase patient morbidity and mortality, and motivate liver transplantation in a significant proportion of patients.

Glycochenodeoxycholic acid (GCDCA) is the major human hydrophobic BA [4] that has been held responsible for cholestasis-associated liver injury [5] [6]. By contrast, another physiological constituent of human bile, ursodeoxycholic acid (UDCA), has been demonstrated to exert anticholestatic effects in several cholestatic disorders and is the treatment of choice in primary biliary cirrhosis, which represents the leading cause of small-duct biliary diseases. The beneficial effect of UDCA has been associated with the inhibition of BA-induced hepatocyte and cholangiocyte apoptosis, and with improved patient survival. However, some considerations should be taken into account about using UDCA in the treatment of CLD. Thus, UDCA is frequently combined or replaced by other therapeutics agents such as corticosteroids, immunosuppressant and other drugs [6] [7].

Nitric oxide (NO) may behave either as a primary mediator of liver damage or as a potent protective agent against injury, depending on its concentration and generating source among other factors [8]. NCX-1000 is a NO-releasing derivative of UDCA that selectively delivers NO to the liver and protects against development of portal hypertension, a common complication of CLD and a leading cause of death in patients with cirrhosis [9]. The endothelial nitric oxide synthase (NOS-3) is the main source of endogenous NO in the liver [10], and its expression may prevent liver damage [11]. Recently, we have shown that transcription factor (TF) SP1 negatively regulates NOS-3 expression during GCDCA-induced cholestasis through direct binding to the NOS-3 promoter (pNOS-3) in an oxidative stress dependent manner. In this regard, specific inhibition of SP1 resulted in the NOS-3 expression increase and the reduction of cell death-related parameters, and was proposed as a new potential target in CLD [12]. Here, we studied the involvement of new proposed TF binding sites (TFBS) for AP-1, GATA-1 and GATA-4 in the regulation of NOS-3 expression during experimental cholestasis in the human hepatocarcinoma cell line HepG2.

## Materials and Methods

### Cell lines and culture conditions

**Fig 1:**
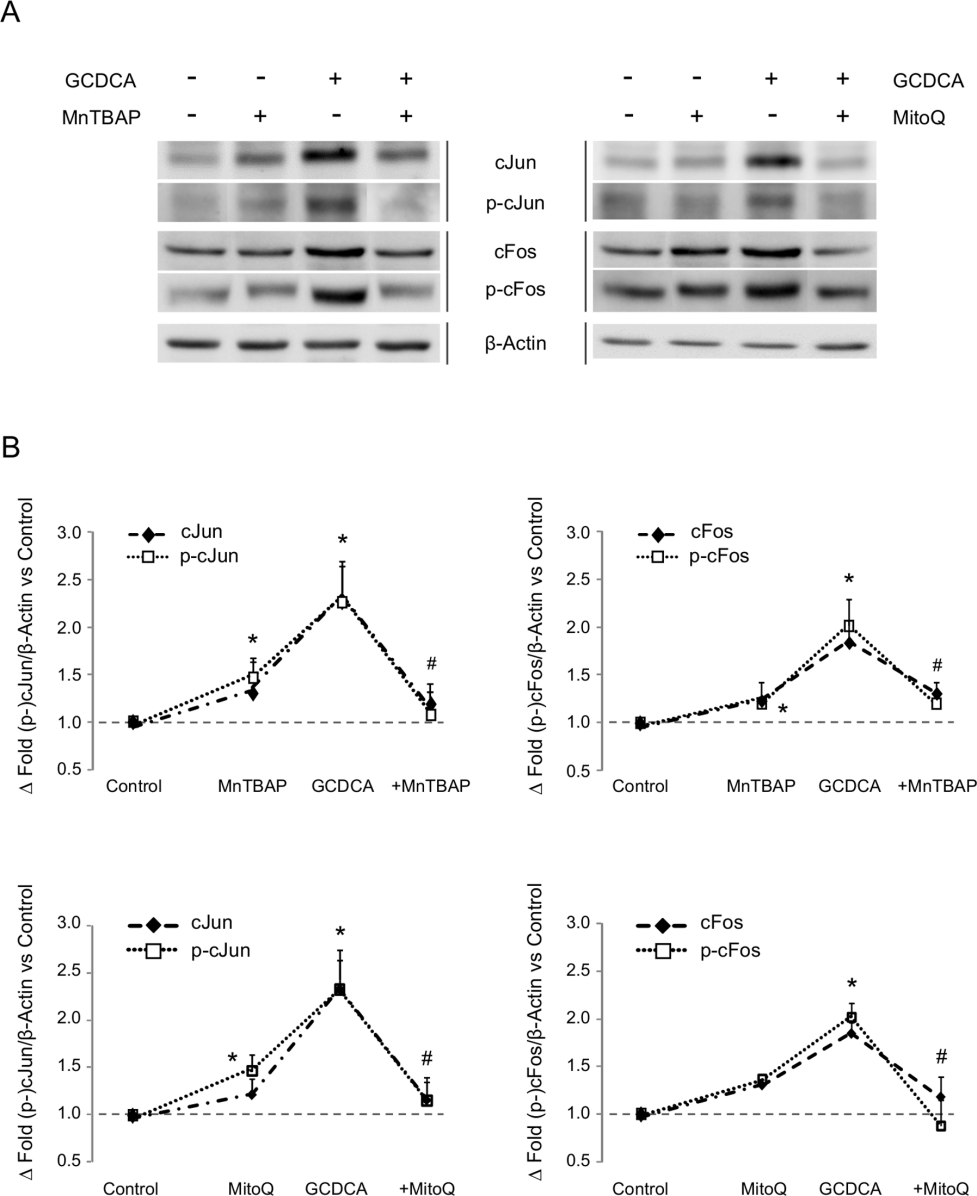
Expression of transcription factors cJun and cFos in GCDCA-treated HepG2. Effect of the administration of MnTBAP or MitoQ. (A) Representative western blots for cJun (n = 3), phospho-cJun (Ser63) (n = 4), cFos (n = 5) and phospho-cFos (Ser374) (n = 4). Β-Actin was used as loading control. (B) Densitometry analysis of blots included in panel A. In the densitometry analysis, the + sign indicates GCDCA administration plus the showed treatment. Data as mean ± SE versus control group. Statistically significant difference versus control group* or versus GCDCA group^#^ are marked.

HepG2 cell line (European Collection of Cell Cultures) was grown in EMEM (Invitrogen-Thermo Fisher Scientific, Massachusetts, USA) supplemented with 1 mM sodium pyruvate and 10% fetal bovine serum (Gibco-Thermo Fisher Scientific, Massachusetts, USA). The pGL4-NOS3 cell line was obtained by stable transfection of HepG2 cells with the plasmid pGL4.20 [luc2/Puro] (Promega, Wisconsin, USA) containing the luciferase reporter gene under the control of the human pNOS-3 (1,601 nucleotides, GenBank accession no. AF387340.1; Fig 1). The control cell line pGL4 was obtained by stable transfection of HepG2 cells with the pGL4.20 [luc2/Puro] vector without promoter sequence insertion [12].

### Experimental procedure

Cells were plated at 150,000 cells/cm^2^ and 0.5 mM GCDCA (Sigma-Aldrich) was administered after 48 h of cell culture. The SOD mimetic MnTBAP (manganese (III) tetrakis (4-benzoic acid) porphyrin chloride) (Calbiochem, Darmstadt, Germany) and the mitochondria-targeted antioxidant MitoQ were administered at concentration of 0.1 mg/ml and 1 μM, respectively. Curcumin (diferuloylmethane), Quercetin (Sigma-Aldrich, Missouri, USA), the NOS inhibitor N^ω^-Nitro-L-arginine methyl ester hydrochloride (L-NAME; Sigma-Aldrich) and the specific AP-1 inhibitor SR 11302 (Santa Cruz Biotechnology, California, USA) were used at 4 μM, 20 μM, 5 mM and 50 μM, respectively. Cells were harvested at different times according to the parameter under study. MitoQ was synthesized according to the procedure described by Kelso et al. [13].

### Growth measurements

Growth capacity and cell viability were determined by plating 4x10^5^ cells on 6 wells plates in 2 ml of the appropriate medium. Cells were incubated at 37°C for 5 days and cell counts were performed at daily intervals by the trypan-blue exclusion test. In order to quantify the number of dead cells accumulated during cell culture, the culture medium was removed and cells were washed with 10 mM PBS. Then, these two volumes were pooled and centrifuged at 2,500 rpm for 5 min, and the resulting pellet joined the pellet obtained after cell trypsinization.

Alternatively, cell proliferation was determined by the 3-(4,5-dimethylthiazol-2-yl)-2,5-diphenyltetrazolium bromide (MTT) assay. Briefly, 14,300 cells were platted on 96 wells plates in 200 μl of culture medium. For the measure, wells were washed with no supplemented cell culture medium and, after the addition of 50 μl MTT (1mg/ml), cells were incubated at 37°C for 2 h. Finally, MTT was carefully removed and insoluble formazan was resuspended in 100 μl DMSO and spectrophotometrically measured at 570 nm with wavelength correction at 650 nm.

### Preparation of cell extracts

Protein extracts from HepG2 were obtained by incubating the cells in a lysis buffer containing 50 mM Hepes, pH 7.5, 100 mM NaCl, 2 mM EDTA, 1% NP-40, 1 mM PMSF, 5 μg/ml aprotinin and 10 μg/ml leupeptin (Sigma-Aldrich). The integrity and concentration of protein samples were determined by the Ponceau staining and the Bradford method, respectively.

### Apoptotic cell death analysis

The apoptosis induction was assessed by the measure of caspase-3 activity in cell extracts as previously described [14]. Briefly, 50 μg or 100 μg of protein were diluted with caspase buffer (50mM Hepes pH 7.5, 100mM NaCl, 10% sucrose, 0.1% CHAPS, 1mM EDTA and 5mM DTT) and the reaction was started with the addition of 100 μM Ac-DEVD-AFC (N-acetyl-Asp-Glu-Val-Asp-7-amino-4-trifluoromethy coumarin) (Bachem AG, Budendorf, Switzerland) in 100 μl of total volume. The fluorescence increase due to enzymatic release of AFC (λ_Ex_ 400nm, λ_Em_ 505nm) was registered every 15 min for 2 h in a GENios Microplate Reader (TECAN). Data were reported as A.U x h^-1^ x mg^-1^ of protein.

### Luciferase reporter assay

The NOS-3 promoter activity was measured in pGL4-NOS3 and pGL4 cells by using the One-Glow Luciferase Assay System (Promega). Briefly, cells were plated in 384-well black microplates (15,000/well) and, after treatment administration, were lysed with 20 μl of 1X Lysis Buffer. 40 μl of cell extract were used for luciferase assay according to the manufacturer´s recommendations.

### Protein expression analysis by Western-Blot

Between 20–100 μg of protein from cell extracts were separated in a 10–12% SDS-PAGE, transferred to nitrocellulose membrane and sequentially blotted against monoclonal or polyclonal primary antibodies. Primary antibodies were anti-NOS3 (sc-654, dilution 1:200), anti-cJun (ref. sc-1694/X, dilution 1:2,000), anti-phospho-cJun (ref. sc-822, diluted 1:300), anti-cFos (ref. sc-52G, diluted 1:200), anti-phospho-cFos (ref. sc-81485, diluted 1:100), anti-phosho-SP1 (ref. ab37707, diluted 1:1,000), anti-cyclin D1 (ref. 2926, diluted 1:2,000) and β-Actin (ref. sc-47778, diluted 1: 10,000). All antibodies were from Santa Cruz Biotechnology (California, USA), except anti-phospho-SP1 (Abcam, Cambridge, UK) and anti-cyclin D1 (Cell Signalling Technology, Massachusetts, USA). The densitometry analysis was performed by Quantity One software (v.4.4.0) (Bio-Rad, California, USA), using β-Actin signal as a loading control.

### Nitric oxide production

NO production was determined by the Griess reaction as we previously described [14]. In order to allow the accumulation of NO-related end products nitrates and nitrites, the cell culture medium was collected 24 h after the administration of the treatment. This ensured the sensitivity and reproducibility of the reaction. Nitrite concentrations were accurately determined by a nitrite calibration curve.

### Identification of Transcription Factors Binding Sites in the pNOS-3

The identification of theoretical TFBS in the pNOS-3 sequence was performed using the three different free online software tools for TFBS prediction: Transcription Factor Search (TFsearch v.1.3; http://www.cbrc.jp.reserach/db/TFSEARCH.html), Transcription Element Search System (TESS; http://www.cbil.upenn.edu/tess) and Transcription Factor Site Scan (TF site scan; http://www.ifti.org/cgi-bin/ifti/Tfsitescan.pl).

### Chromatin Immunoprecipitation (ChIP)–RT-qPCR assay

ChIP-assays were performed as described in [15], using isolated nuclei from the formaldehyde-cross-linked HepG2 cells. Immunoprecipitation was performed using primary antibodies anti-cJun or anti-cFos, and magnetic beads (Dynabeads® Protein G, Life Technologies). RNA polymerase II (PolII) and normal rabbit IgG were used as positive and negative control, respectively, using antibodies anti-PolII (ref. sc-899) and anti-IgG (ref. sc-2027). 4 μg of antibody was used for each experiment. All primary antibodies were from Santa Cruz Biotechnology. Purified samples were analysed by RT-qPCR, using SensiFast SYBR kit (Bioline, London, UK), and primers used to detect target sequences were as follows: -666 AP-1 site, 5’- CTTTTGTGTCCCCCACTTGAG -3’ and 5’- CAATTTCCTGGAACCCCCAC -3’; -235 GATA-1/-4 site, 5’- AGGGCTCTGCTGGACACCT -3’ and 5’- GCTGTGAGGACTGAGGCTGAT -3’. Primers to detect the NOS-3 (NM_000603.4) and glyceraldehyde 3-phosphate dehydrogenase (GAPDH; NM_002046.4) coding regions are summarized in [12]. For quantification purposes, a calibration curve was elaborated for each amplicon by 10-fold serial dilutions of the total input sample. Results were expressed as percent of input considering the input as 100% and the negative control as 0%.

### Statistical Analysis

Results were expressed as mean ± standard error of a minimum of 3 independent experiments. Data were compared by using the Kruskal-Wallis non-parametric method, and the Dunnett's T3 test for post-hoc multiple comparison analysis. Data from ChIP analysis, involving control and GCDCA-treated samples, were analysed by using the Mann-Whitney U test. Statistical comparisons were two-tailed, and significance was defined as *p*<0.05. All tests and calculations were done with the statistical package StatView 5.0 (SAS Institute, Inc.) and SPSS 15.0 (IBM) for Windows.

## Results

### GCDCA-induced oxidative stress stimulates the expression and activation of cJun and cFos

Bioinformatic analysis of the pNOS-3 sequence identified candidate TFBS involved in the modulation of the NOS-3 expression. Only those sites identified by all the three software tools were selected for further analysis. We ignored the binding sites proposed for SP1, which were previously analysed in depth in the study by González-Rubio et al. [12]. Therefore, we considered TFBS for AP-1 and GATA-1 at positions described in Table 1.

**Table 1:** Proposed transcription factor binding sites in the pNOS-3 region (1.6 Kb) for AP-1 and GATA-1. TFSEARCH, TESS and TF SITE SCAN are free online software tools for the prediction of transcription factor binding sites. ‘Position’ makes reference to the pNOS-3 sequence (NCBI accession number GI: 14669582). W is A/T, M is A/C, S is C/G, K is G/T, R is A/G.

	TFSEARCH		TESS		TFSITESCAN	
TF	Position	Sequence	Position	Sequence	Position	Sequence
**AP-1**	-666	TTGAGTCAT	-665	TGAGTCA	-665	TGAGTCA
**GATA-1**	-235	CCACTTATCAGCCT	-232	CTTATCA	-232	WGATAMS

To elucidate whether AP-1 (proteins cFos and cJun as constituent elements) was involved in the NOS-3 regulation during cytotoxicity mediated by GCDCA, we determined AP-1 expression and activation rates in HepG2 cells. GCDCA increased the expression of proteins cJun (233%, p = 0.024) and cFos (185%, p = 0.006), as well as their phosphorylation states (233% p = 0.035, 202% p = 0.009, and 200% p = 0.003, respectively). Cellular protection against oxidative injury was promoted by treatment with the antioxidants MnTBAP or mitoQ [12], which in turn reduced the expression and phosphorylation rates of TFs cJun and cFos during GCDCA-induced cytotoxicity (Fig 1).

### GCDCA increases cJun/cFos binding to pNOS-3

**Fig 2:**
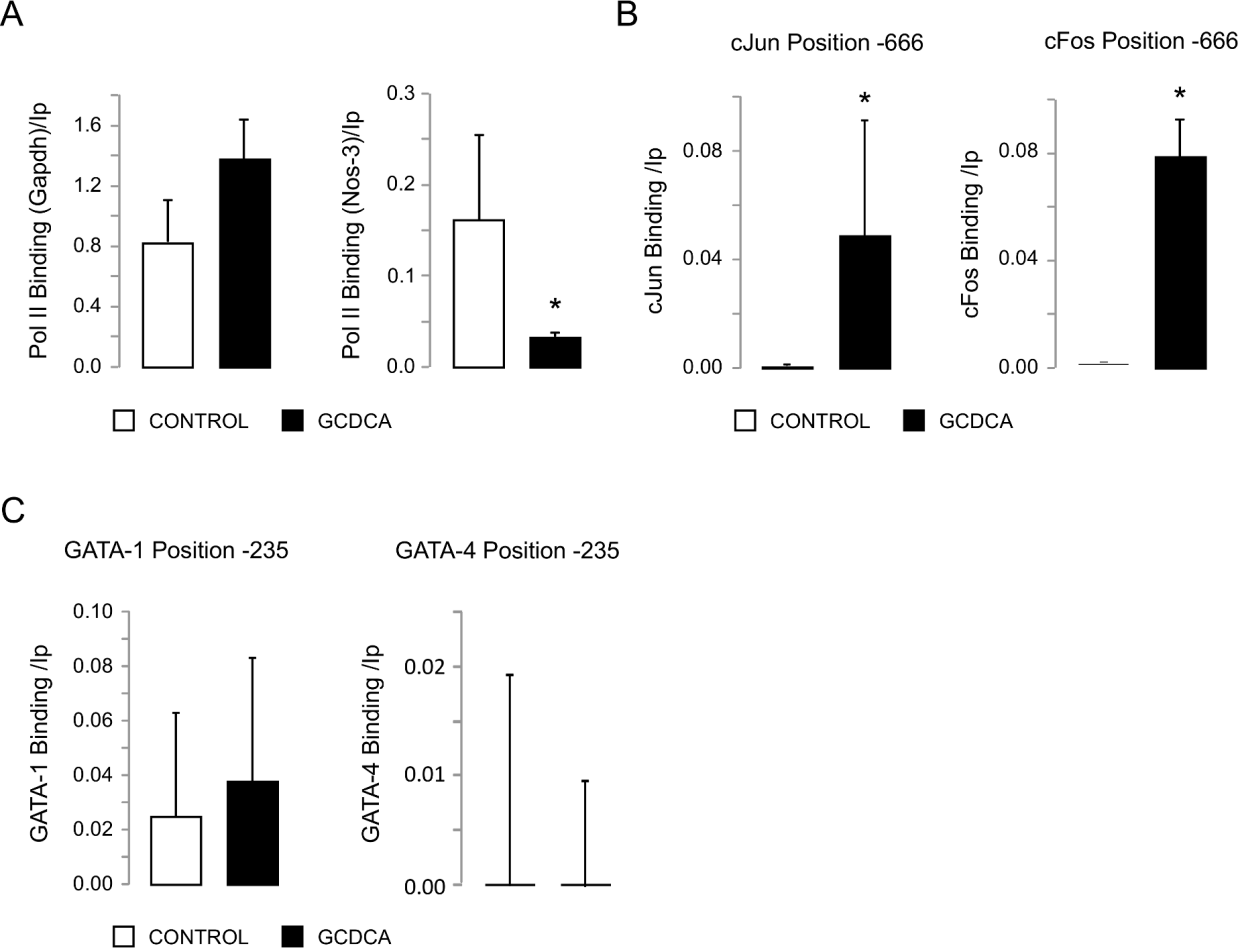
Transcription factors cJun and cFos bind to the NOS-3 promoter after GCDCA administration. (A) Gene expression of GAPDH and NOS-3 as positive controls. (B) Enrichment of cJun and cFos binding to the NOS-3 promoter (position -666) after GCDCA administration. (C) Evaluation of the binding of GATA-1 and GATA-4 to the NOS-3 promoter (position -235) after GCDCA administration. Data expressed as mean ± SE of three independents experiments. Statistically significant difference versus control group (no GCDCA) is marked*.

In order to test the effect of GCDCA administration on the AP-1 binding capacity to the pNOS-3 sequence, we performed ChIP assays. We selected a TFBS for AP-1 located at position -666 (Table 1). GAPDH and NOS-3 were used as positive controls of immunoprecipitation and gene expression, respectively (Fig 2A). Immunoprecipitation of samples from HepG2 cells with antibodies anti-cJun or anti-cFos showed binding of cJun (p = 0.021) and cFos (p = 0.034) at this position of the pNOS-3 after GCDCA administration (Fig 2B). Additionally, we tested the binding of TFs GATA-1 and GATA-4 at position -235 of the pNOS-3 (Table 1). However, GATA-4 did not bind to position -235 of pNOS-3 neither at basal conditions, nor after GCDCA administration. In addition, no significant differences were observed after GCDCA administration when we studied the binding of GATA-1 to the pNOS-3 (Fig 2C).

### AP-1 participates in NOS-3 downregulation during GCDCA-induced cytotoxicity

**Fig 3:**
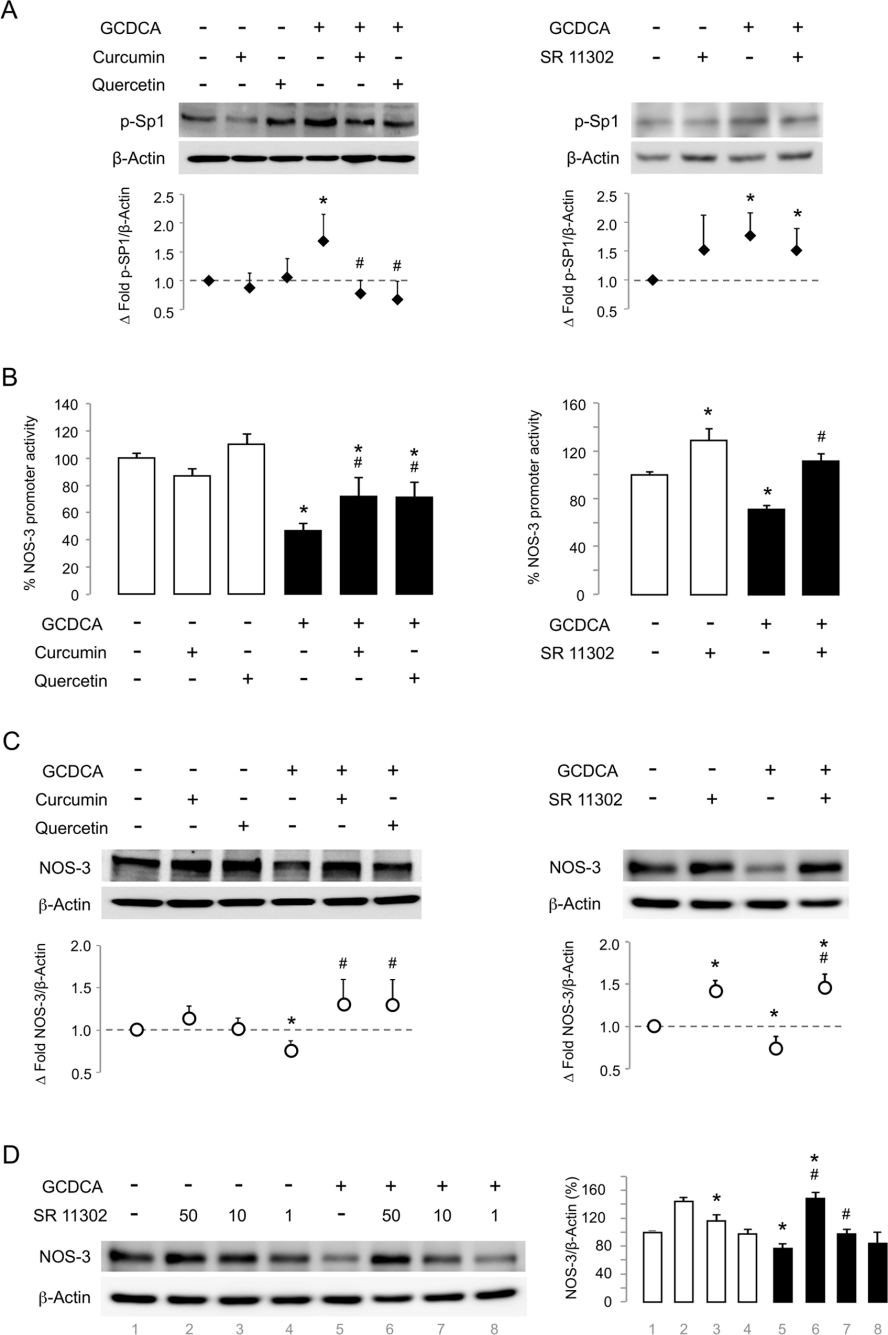
AP-1 downregulates NOS-3 expression during cytotoxicity by GCDCA. Cells not exposed (control group) / exposed to GCDCA (GCDCA group) were treated with Curcumin, Quercetin or the synthetic retinoid SR 11302. (A) Representative western blot for phospho-SP1 (Thr453) (Curcumin and Quercetin, n = 3; SR 11302, n = 7). (B) NOS-3 promoter activity (Curcumin and Quercetin, n = 6; SR 11302, n = 3). (C) NOS-3 protein expression (n = 3). (D) Dose-dependent NOS-3 expression recovery by SR 11302 during GCDCA cytotoxicity. SR 11302 was assayed at 50, 10 and 1 μM. When no indicated, SR 11302 was used at 50 μM. Densitometry analysis for panels (A), (C) and (D) are shown. Data expressed as mean ± SE versus control group. Statistically significant difference versus Control group* or versus GCDCA group^#^ are marked.

Then, we evaluated the involvement of AP-1 in the NOS-3 regulation during GCDCA-induced cell death by testing the TF inhibitors (TFIs) curcumin and quercetin. However, since these two compounds affected the phosphorylation state of the TF SP1 (Fig 3A), we also used the synthetic retinoid SR 11302 that is considered a specific inhibitor of AP-1. As Fig 3A shows, no significant effect on SP1 phosphorylation was detected when SR 11302 was used.

When we later investigated the effect of AP-1 inhibition in GCDCA-treated pGL4-NOS3 cells, we found that all assayed TFIs, curcumin, quercetin and the specific AP-1 inhibitor SR 11302, were able to restore the pNOS-3 transcriptional activity (Fig 3B). These findings were consistent with the NOS-3 protein expression increase (Fig 3C). Thus, the highest concentration of SR 11302 caused an increase of 148% in the NOS-3 expression during GCDCA administration when compared to control group (Fig 3D).

### Specific inhibition of AP-1 protects against the bile acid-induced apoptotic cell death

**Fig 4:**
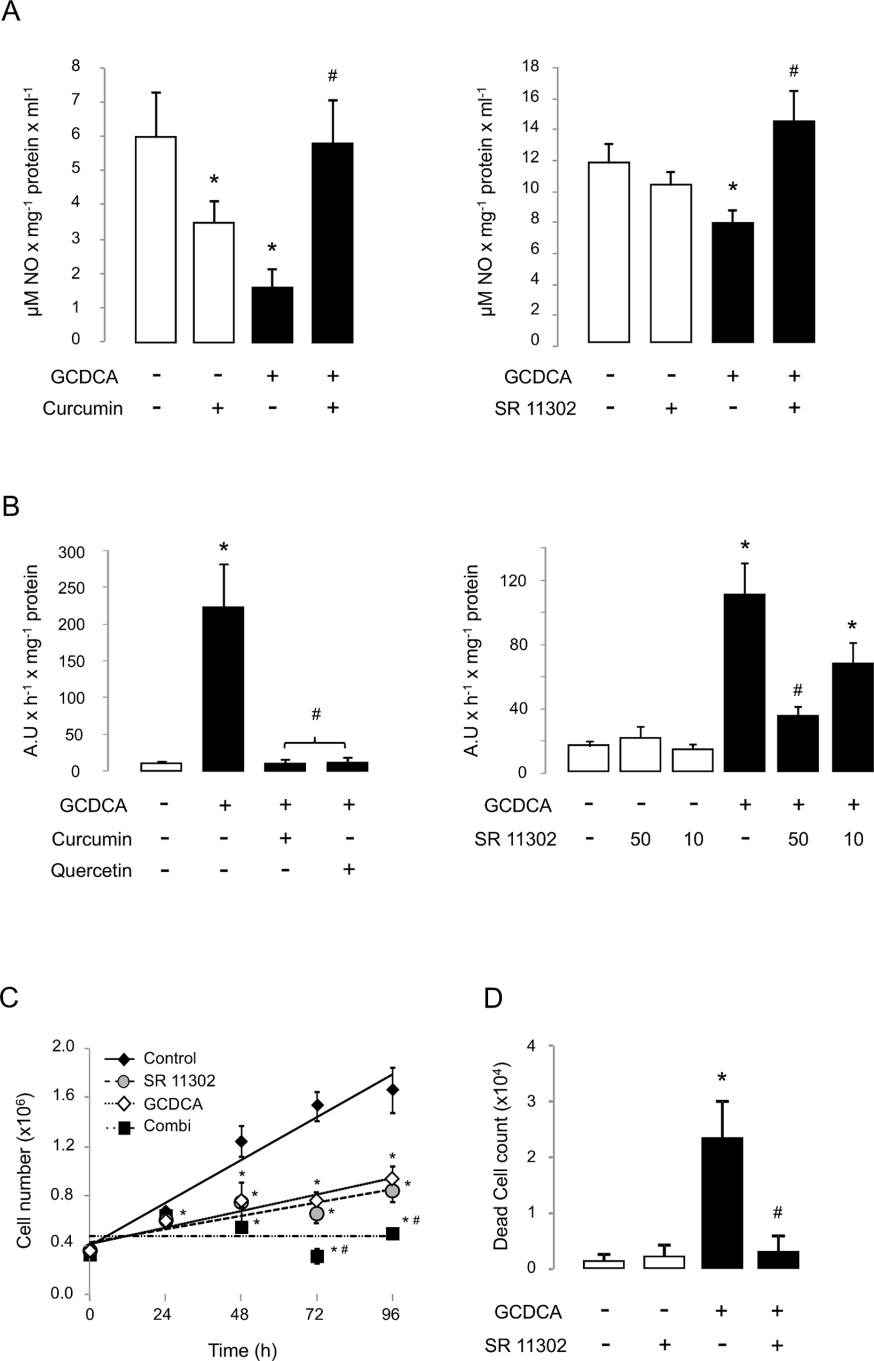
NOS-3 activity recovery by AP-1 inhibition is associated with cell survival during experimental cholestasis. Effects of AP-1 inhibition on (A) accumulation of NO-end products (n = 3), (B) caspase-3-associated activity (n = 4), (C) cell proliferation and (D) dead cell count (n = 3). In panel (B), SR 11302 was assayed at 50 and 10 μM. When no indicated, SR 11302 was used at 50 μM. Data expressed as mean ± SE. Statistically significant difference versus control group* or versus GCDCA group^#^ are marked.

As shown in Fig 4, the NOS-3 expression recovery in GCDCA-treated HepG2 cells by the specific inhibition of AP-1 was related to a higher extracellular accumulation of NO-oxidation end products (Fig 4A), and associated with a dose-dependent reduction on caspase-3 activity (Fig 4B), which is involved in the activation cascade of caspases responsible for apoptosis. These results are consistent with those shown for the non-specific AP-1 inhibition with curcumin or quercetin (Fig 4A and 4B). However, when we later analyse the cell doubling time after 96 hours of culture, it was 41.7±3.3 hours at basal condition, 72.6±7.3 hours with SR 11302 (p<0.05), 65.0±9.1 hours with GCDCA (p<0.05) and 160.7±17.0 hours after GCDCA plus SR 11302 administration (p<0.05) (Fig 4C). Thus, although the specific inhibition of AP-1 resulted in a drop of the duplication rate of the human hepatocarcinoma cell line HepG2, this was not associated with an increased caspase-3 activity (Fig 4B) or with cell death. Moreover, the higher concentration of SR 11302 significantly reduced the number of accumulated dead cells during cytotoxicity induced by GCDCA (Fig 4D).

**Fig 5:**
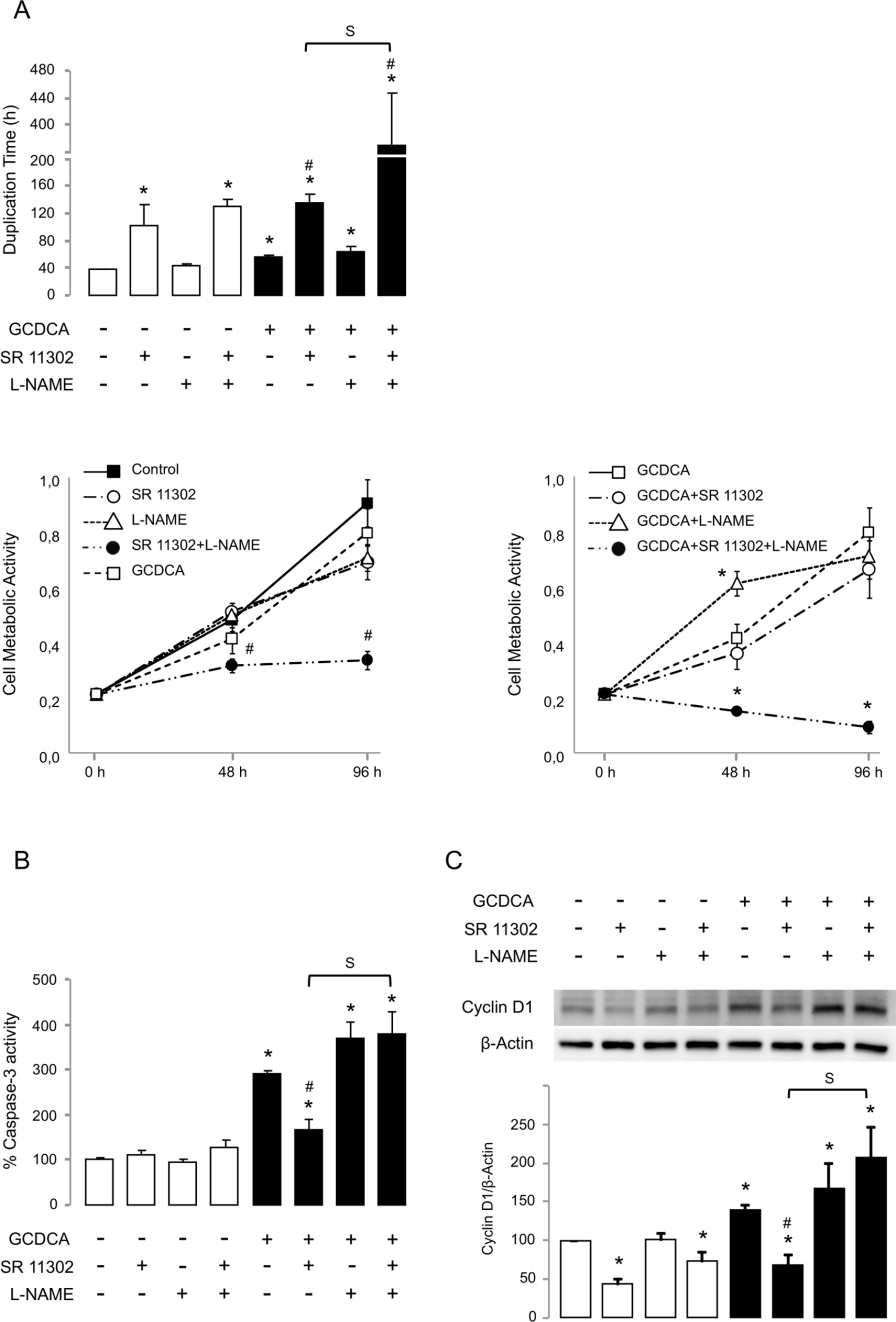
NOS inhibition by L-NAME suppresses cell protection by SR 11302 during cholestasis. Effect of inhibition of NOS activity on (A) cell doubling time (n = 6) and cell metabolic activity (n = 8); (B) caspase-3-associated activity (n = 3); and (C) cyclin D1 expression (n = 4). SR 11302 was used at 50 μM. In panel (A), only positive data for cell doubling time were used (n = 4 for group ‘GCDCA + SR 11302’, and n = 3 for group ‘GCDCA + SR 11302 + L-NAME’). Data expressed as mean ± SE. Statistically significant difference versus control group* or versus GCDCA group^#^ or between groups ‘GCDCA + SR 11302’ and ‘GCDCA + SR 11302 + L-NAME’ (S) are marked.

In order to determine the involvement of NOS-3 expression recovery in the anti-apoptotic effect of SR 11302, we used the NOS inhibitor L-NAME. When it was combined with the retinoid, L-NAME inhibited the protective effect of SR 11302 during GCDCA cytotoxicity (Fig 5). Thus, while the NOS inhibitor *per se* had no effect on cell proliferation (Fig 5A) and cell death (Fig 5B), it significantly inhibited the caspase-3 activity decrease induced by SR 11302 during cholestasis (Fig 5B). L-NAME did also cause a cell doubling time increase in these conditions (Fig 5A). Similar results were obtained when we assessed the cell metabolic activity by the MTT assay (Fig 5A).

The antiproliferative capability of SR 11302 was related to the expression inhibition of cyclin D1, which is involved in the cell cycle progression (Fig 5C). Surprisingly, the toxic bile acid GCDCA induced the expression of cyclin D1 and not its inhibition as Fig 5A suggested. Under such circumstances, L-NAME inhibited the cyclin D1 expression decrease induced by the retinoid, just as previously noted for caspase-3 activity.

## Discussion

It has been previously reported that NOS-3 expression in the liver has a protective role. Different conditions involved in the generation of oxidative stress may regulate the pNOS-3 activity. Cholestasis is a disorder that is associated with oxidative damage and with the NOS-3 expression deregulation. In the model used in this study, GCDCA administration has been associated with a failure in the electron flow between respiratory complexes II and III, which is considered the main source of ROS generation during bile acid-induced toxicity. Recently, we have shown evidence about the involvement of SP1 in the oxidative stress-dependent regulation of pNOS-3 activity during GCDCA-induced cytotoxicity. Thus, SP1 negatively regulates the transcriptional activity of pNOS-3 and NOS-3 expression and activity [12].

In the present study, we have identified new TFBS candidates in the pNOS-3 sequence, which may be involved in the gene expression regulation by BAs. Among them, the binding site for AP-1 was particularly interesting because, as we previously noted for SP1, it is a redox-sensitive TF involved in a wide spectrum of cellular processes, including the NOS-3 expression regulation [16] and the BA-induced cell apoptosis [17]. Cellular response to GCDCA was characterised by an increased expression and activation of cJun and cFos, and increased binding of these factors to the TFBS identified within the pNOS-3 at position -666. These findings suggest that AP-1 complex binds at this position as a heterodimer, resulting in a decrease of pNOS-3 activity and NOS-3 protein expression. The administration of curcumin and quercetin as AP-1 inhibitors [18, 19] [19] reverted the NOS-3 expression decrease. However, since these two compounds have also been described as SP1 inhibitors [20] [21] with antioxidant properties [22] [23], and both, SP1 and oxidative stress, are related to NOS-3 expression regulation during cholestasis [12], these results could not be directly attributed to the inhibition of AP-1. As expected, the specific AP-1 inhibition by SR 11302 resulted in cell protection against GCDCA-induced cell death through the same mechanism previously described for antioxidant treatment or the SP1 inhibition. Thus, the retinoid recovered pNOS-3 activity and NOS-3 expression by inhibiting the AP-1 activity.

It has been reported that AP-1 is involved in NOS-3 up-regulation by cyclosporine A [24] or hypoxia [25], which are related to oxidative stress [26] [27]. Conversely, the antioxidant molecule nordihydroguaiaretic acid up-regulates NOS-3 expression through increase of AP-1 binding to the pNOS-3 in pulmonary arterial endothelial cells [28]. Kumar et al. demonstrated that AP-1 binding at the same pNOS-3 position reported in the present study regulated the promoter activity in response to oxidative stress [29]. However, in that report, the addition of hydrogen peroxide decreased the pNOS-3 activity by reducing AP-1 binding to the pNOS-3. Here, we also observed a promoter activity decrease as a consequence of higher intracellular production of ROS, but it was associated with an increase of AP-1 binding at that promoter position. This observation agrees with previous reports about the role of SP1 as transcriptional repressor of NOS-3 expression in the same cellular model of cholestasis [12]. In this regard, it is striking that AP-1 regulates numerous mammalian genes often in cooperation with TFs such as SP1. Interestingly, the AP-1 activity on the pNOS-3 was dependent on the presence of SP1, being both factors necessary to trigger pNOS-3 activation [30].

GATA binding to the pNOS-3 at position -235 was required to modulate SP1 activity during basal promoter activity [31]. Since the bioinformatic analysis identified a TFBS for GATA-1 in the pNOS-3 sequence, and GATA-4 binding at position -235 has been related to the pNOS-3 activity regulation [32], we additionally tested GATA-1 and GATA-4 binding at this position. However, we observed no promoter binding for GATA-4 or no binding differences for GATA-1 after GCDCA administration. Thus, if GATA binding to the pNOS-3 at position -235 was required to modulate SP1 activity during basal promoter activity, it did not involve GATA-4.

Our data illustrated that AP-1 may function as transcriptional repressor of NOS-3 gene expression through direct binding to the promoter sequence at position -666. To our knowledge, this is the first study that identifies an inhibitory role for AP-1 in the NOS-3 expression. However, this is not an exceptional case since overexpression of cJun and cFos in epithelial cells has been previously associated with a reduction of NOS-2 promoter activity [33]. Oxidative stress induced by GCDCA caused the activation of TFs cJun and cFos, and their binding to the pNOS-3 at position -666. This fact was related to the repression of promoter activity, the NOS-3 expression/activity decrease and cell death. In a model of experimental cholestasis *in vivo*, the observed hepatocellular damage was associated with the inhibition of NOS-3 expression and the activation of TFs cJun and cFos (data not shown). The regulation of cJun and cFos activities by oxidative stress and the involvement of NOS-3 in this process represent a new mechanism by which the accumulation of BAs causes a cytotoxic effect, and provides a new potential therapeutic target for CLD.

The biological activities of retinoids are believed to be mediated by transcriptional activation of retinoic acid response element (RARE) and inhibition of AP-1 activity. In cholestatic diseases, the addition of retinoic acid to UDCA might be an effective supplemental therapy with UDCA [34]. However, clinical use of retinoids is hampered by their adverse safety profile [35], which could be due to transcriptional activation of RARE. Thus, only AP-1 transrepression, but not RARE transactivation mediated by retinoids appears to be causally related to their antitumor activities [36]. Fortunately, a subset of synthetic retinoids are able to inhibit AP-1 activity without activating the transcription of RARE [36] [37], which have been previously proposed as therapeutic agents with reduced side effects. Thus, these retinoids are transcriptionally inactive and have lost the differentiating effects of retinoic acid, while retaining their antiproliferative properties, which come from the inhibition of AP-1 activity. SR 11302 belongs to this new class of retinoids and its use has been previously reported in human pathologies such as peritoneal inflammation [38] and cancer [36] [39]. In our experimental model of cholestasis, the inhibition of AP-1 by pharmacological blockade with SR 11302 was associated with protection from GCDCA-induced cell death by inhibiting caspase-3 activity. This is consistent with the previously reported study about the important role of apoptosis in the pathogenesis of liver injury during cholestasis, and the involvement of AP-1 upregulation in this process [17]. The administration of the commonly used NOS inhibitor L-NAME did inhibit the anti-apoptotic effect of SR 11302 during GCDCA treatment. This, together with the fact that no expression of NOS-2 was detected in our experimental model of cholestasis, suggests that NOS-3 regulation by AP-1 is a key process involved in the hepatocellular damage during cholestasis.

Although AP-1 activity can antagonize apoptosis in some tumor types such as liver tumors [40], and genes that encode Bcl2 family members have been proposed as anti-apoptotic targets regulated by the TF [41], inhibition of AP-1 by SR 11302 in HepG2 cells did not induce caspase-3 activity and it was not associated with the accumulation of dead cells. However, SR 11302 was related to the alteration of cell proliferation, which is consistent with its well-known antitumor capacity [36] [39]. At basal condition, the antiproliferative effect of SR 11302 was related to the expression inhibition of cyclin D1, a member of the cyclin family that is required for progression through the G1 phase of the cell cycle. However, although cyclin D1 promotes cell growth, overexpression of cyclin D1 can lead to premature G1-S phase transition and apoptosis [42]. In cholestasis, cyclin D1 overexpression has been implicated in toxic bile acid-induced Bax translocation, cytochrome c release and apoptosis of hepatocytes [43] [44]. Accordingly, the apoptosis induction by GCDCA was associated with the expression increase of cyclin D1. The inhibition of cyclin D1 expression by SR 11302 in our experimental model of cholestasis increased the cell doubling time (decreasing cell metabolic activity) but it significantly reduced the caspase-3 associated activity. Since NOS-3 expression was inhibited by GCDCA, the administration of L-NAME had no effect on cyclin D1 expression or cell proliferation. However, the NOS inhibitor counteracted the protective role of SR 11302, which is associated with the NOS-3 expression recovery. Thus, in the presence of L-NAME, the retinoid failed to reduce cyclin D1 expression and caspase-3 activity in GCDCA-treated HepG2 cells.

Taken together, our findings relate the toxic effects of bile acids with cyclin D1 overexpression through the NOS-3 expression inhibition by AP-1 (S1 Fig). The apparent safety of SR 11302 compared with other candidate agents such as mithramycin A or tolfenamic acid [12], makes the synthetic retinoid an interesting alternative therapeutic approach for CLD.

## Supporting Information

S1 Fig NOS-3 expression inhibition by AP-1 is required to bile acid-induced cytotoxicity and is associated with cyclin D1 overexpression. GCDCA induces cell death by oxidative stress-dependent AP-1 expression increase, NOS-3 downregulation and cyclin D1 overexpression. Antioxidant treatment inhibits AP-1 upregulation and cell death. AP-1 inhibition by SR 11302 reduces cell death by increasing NOS-3 expression/activity. NOS-3 activity inhibition by L-NAME is related to cyclin D1 expression increase and cell death.
(TIF)
