# Supplementary figures and images for: AP-1 Inhibition by SR 11302 Protects Human Hepatoma HepG2 Cells from Bile Acid-Induced Cytotoxicity by Restoring the NOS-3 Expression
Source: PLoS One. 2016 Aug 4;11(8):e0160525. doi: 10.1371/journal.pone.0160525 (PMC4973998; doi:10.1371/journal.pone.0160525)

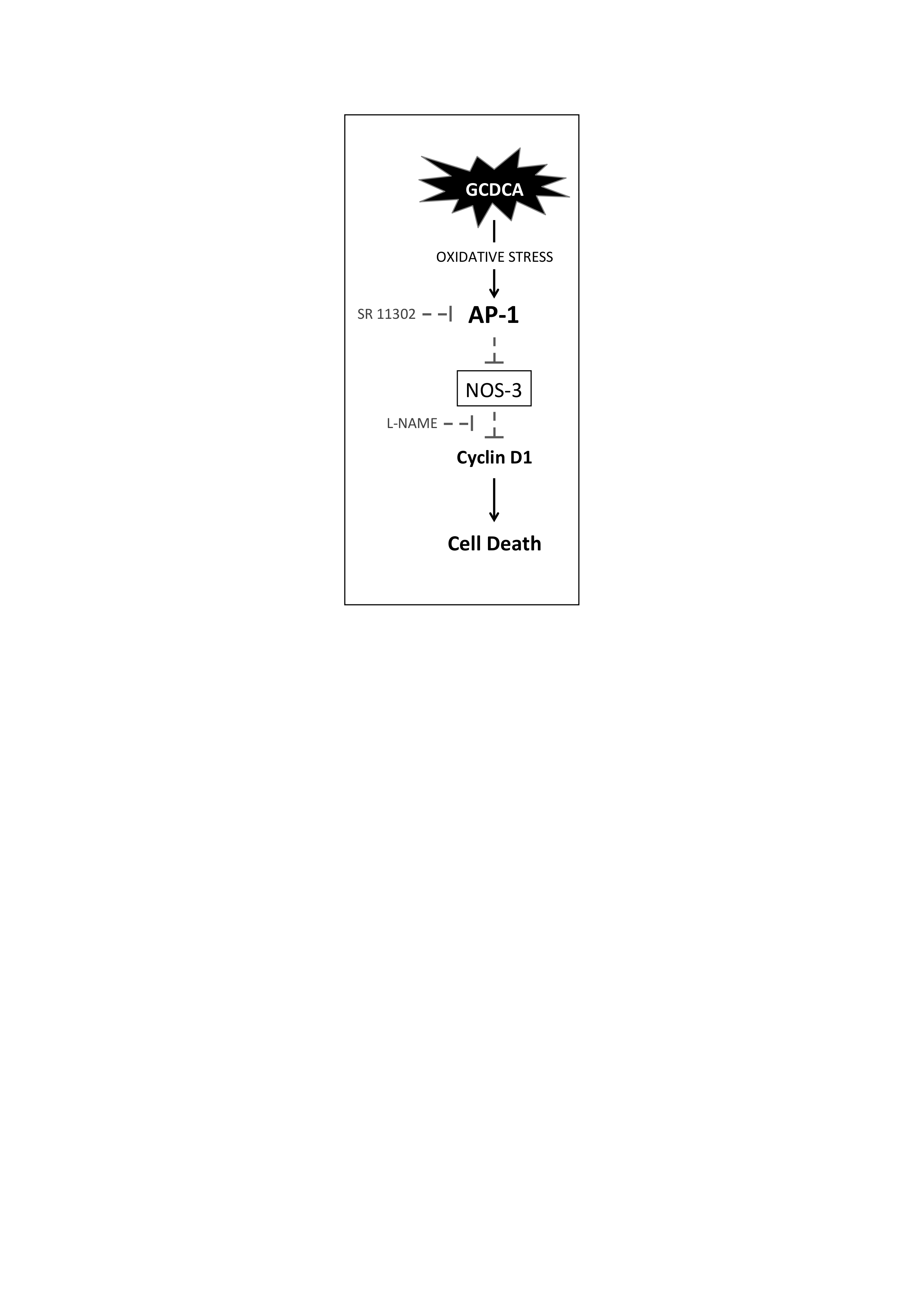

Supplement: S1 Fig — GCDCA induces cell death by oxidative stress-dependent AP-1 expression increase, NOS-3 downregulation and cyclin D1 overexpression. Antioxidant treatment inhibits AP-1 upregulation and cell death. AP-1 inhibition by SR 11302 reduces cell death by increasing NOS-3 expression/activity. NOS-3 activity inhibition by L-NAME is related to cyclin D1 expression increase and cell death. (TIF) [file pone.0160525.s001.tif]
